# Fish Buffet: Dietary Observations in the Oman Saw‐Scaled Viper (*Echis omanensis*), with a Review of Fish‐Eating Vipers

**DOI:** 10.1002/ece3.74371

**Published:** 2026-09-24

**Authors:** Theo Busschau, Arabella Willing, Borja de las Heras, Rami Khashab, Abdul Nasser Obeidat, Altaf Habib, Andrew Gardner

**Affiliations:** ^1^ New York University Abu Dhabi Abu Dhabi UAE; ^2^ Emirates Nature – WWF Abu Dhabi UAE; ^3^ Independent Researcher Copenhagen Denmark; ^4^ School of Arts and Sciences Lebanese International University Matn Lebanon; ^5^ Fujairah Environment Authority Fujairah UAE

**Keywords:** citizen science, natural history observations, piscivory, snakes, Viperidae

## Abstract

Diet is an important ecological variable that has contributed significantly to the evolution of snakes, yet the diets of many species are still poorly known. Here, we report for the first time observations of the arid‐adapted *Echis omanensis* feeding on fish. On two occasions we observed adults of this species preying on *Garra barreimiae* in drying pools in the United Arab Emirates. In one case, a snake was seen ingesting a fish in Wadi Shawka, Ras Al Khaimah, and in another, an individual was observed actively searching for and capturing fish in Wadi Wurrayah, Fujairah. These records provide direct evidence that *E. omanensis* can opportunistically exploit fish as prey in ephemeral freshwater habitats. To place these observations in context, we reviewed primary and non‐primary sources to identify 14 fish‐eating species in the Viperidae family. This review alludes to fish as rare, seasonal or region‐specific prey among vipers. More broadly, these findings underscore the value of natural history observations for documenting unusual prey in snakes.

## Graphical abstract

We present the first records of the arid‐adapted Oman saw‐scaled vipers feeding on fish. Apon further review we identified 14 fish‐eating vipers globally. These findings suggest that fish may be more common in the diets of vipers than realized, although still rare, and likely to be region‐ or season‐specific prey.

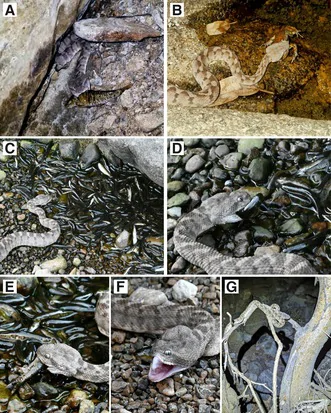

The diets of snakes are important ecological variables that give insight into the evolution of species and how they interact with their environment. Among vipers (venomous snakes in the Viperidae family), feeding ecology has contributed significantly to the evolution of fang and skull morphology (Holding et al. 2022; Carrasco et al. 2023), and the evolution of specialized phenotypes related to ambush foraging, including prey‐specific luring behavior (del Mármol et al. 2016; Glaudas and Alexander 2017) and visual as well as chemical crypsis (Miller et al. 2015; Pizzigalli et al. 2020). Diet has also been linked to variation in venom composition (Barlow et al. 2009; Richards et al. 2012; Holding et al. 2021; Op den Brouw et al. 2021). It has therefore been suggested that inter‐ and intraspecific variation in the diets of venomous snakes may have indirect effects on the consequences and treatment of snake bites in humans (Casewell et al. 2020). Despite the ecological and potential epidemiological importance, the diets of many snake species are still poorly known and often limited to a few published records or inferred from closely related species (see Maritz et al. 2021). Herein, we document, the first natural observations of *Echis omanensis* feeding on fish, a rather peculiar prey choice for a snake in an arid‐adapted genus and largely terrestrial ambush foraging family. We further reviewed reports of fish in the diets of vipers and to what extent these reports can be verified by primary sources (natural history observations or quantitative gut content studies).

*Echis* comprises 12 recognized species distributed across the deserts, shrubland, and arid savannas of North, East, and West Africa to South and West Asia (Kaviani et al. 2023). Like many vipers, *Echis* are opportunistic hunters that feed on a variety of prey (Glaudas et al. 2019), including vertebrates and invertebrates (Barlow et al. 2009). The variety and abundance of prey species can be low in arid environments and therefore the generalist diets of *Echis* are likely to have contributed to their successful adaptation. Yet there is a considerable degree of variation in the dietary composition across species (Barlow et al. 2009), where the diets of the *E. pyramidium* group (including *E. pyramidum* , *E. leucogaster* , and *E. khosatzkii* ) and the *E. carinatus* group (including *E. carinatus* and *E. multisquamatus*) comprise mostly invertebrates (> 50%) and the diets of the *E. ocellatus* group ( *E. ocellatus* and *E. jogeri* ) comprise mostly vertebrates (> 50%). In contrast, the *E. coloratus* group ( *E. coloratus* and *E. omanensis* ) feed almost exclusively on vertebrate prey, including frogs, reptiles, and mammals, while invertebrates accounted for only 7.5% of their observed diet.

The varied diets of *Echis* species correlate with venom variation, suggesting that venom evolution is driven by selection for different diets (Barlow et al. 2009). It is not clear what factors influence diet, but it is likely that diet, and therefore venom evolution, is driven by the most abundant appropriately sized prey available to each species/population where they occur. In addition to the frogs, reptiles, and mammals observed in museum specimens from the *E. coloratus* group, *E. coloratus* has previously also been observed feeding on migratory birds at a desert oasis (Tsairi and Bouskila 2004), alluding to the hypothesis that the diet composition of *E. coloratus* may be determined by the seasonal availability of prey. In the description of *Echis omanensis* , distinguishing the populations from the mountains of the United Arab Emirates and Northern Oman from *E. coloratus*, known from southern and western Arabia, Babocsay (2004) noted that the diet of *E. omanensis* is likely to be similar to the diet of *E. coloratus* and that both species may feed on a variety of vertebrates.

Despite the lack of published dietary information for *E. omanensis* , its diet is often reported to mirror the known diet of *E. coloratus* , including mammals, reptiles, frogs, birds and invertebrates (Egan 2008; Gardner 2013; Burriel‐Carranza et al. 2022). Egan (2008) noted that *E. omanensis* also feeds on fish, based on their willingness to accept fish in captivity (Egan, personal communication). Feulner (2023) reported that *E. omanensis* hunts in dry riverbeds (locally referred to as wadis) at the water's edge where toads are the most abundant vertebrate prey and suggested that *E. omanensis* may also prey on tadpoles and fish trapped in drying pools. Feulner (personal communication) recalls observing a young *E. omanensis* (approximately 30 cm in length) around the mid‐1990's at a drying pool in Wadi Wurrayah, Fujairah, United Arab Emirates, where it was loosely coiled around three or four Oman garra fish ( *Garra barreimiae* ). While he did not observe ingestion, it seemed clear that the viper was hunting the fish. Our observations of *E. omanensis* consuming fish in the wild confirm these observations.

*Observation 1*. On 23 May 2023, in Wadi Shawka, Ras Al Khaimah, United Arab Emirates, at 7:10 pm, we observed an adult *E. omanensis* with a subdued *G. barreimiae* in its mouth (Figure 1A). At the time of observation, the snake had most of its body concealed within a crevice between boulders, with approximately one‐third exposed and positioned about 30 cm from the water's edge. The snake was already holding the fish in its mouth when we saw it and shortly after initiated ingestion. The swallowing process lasted around 1 min. The section of the wadi where this observation occurred had low water levels, causing high densities of *G. barreimiae* to congregate in the small pools alongside several Arabian toads ( *Sclerophrys arabica* ). We did not observe the snake catching the fish in this observation, but at this particular pool the fish were still able to swim and often close to the surface due to their high density. We assume the viper had to have caught the fish when it was swimming close to the surface at the edge of the pool. On a previous occasion, we observed *E. omanensis* feeding on a toad, *S. arabica* , at the water's edge in the same wadi (Figure 1B).

**FIGURE 1:**
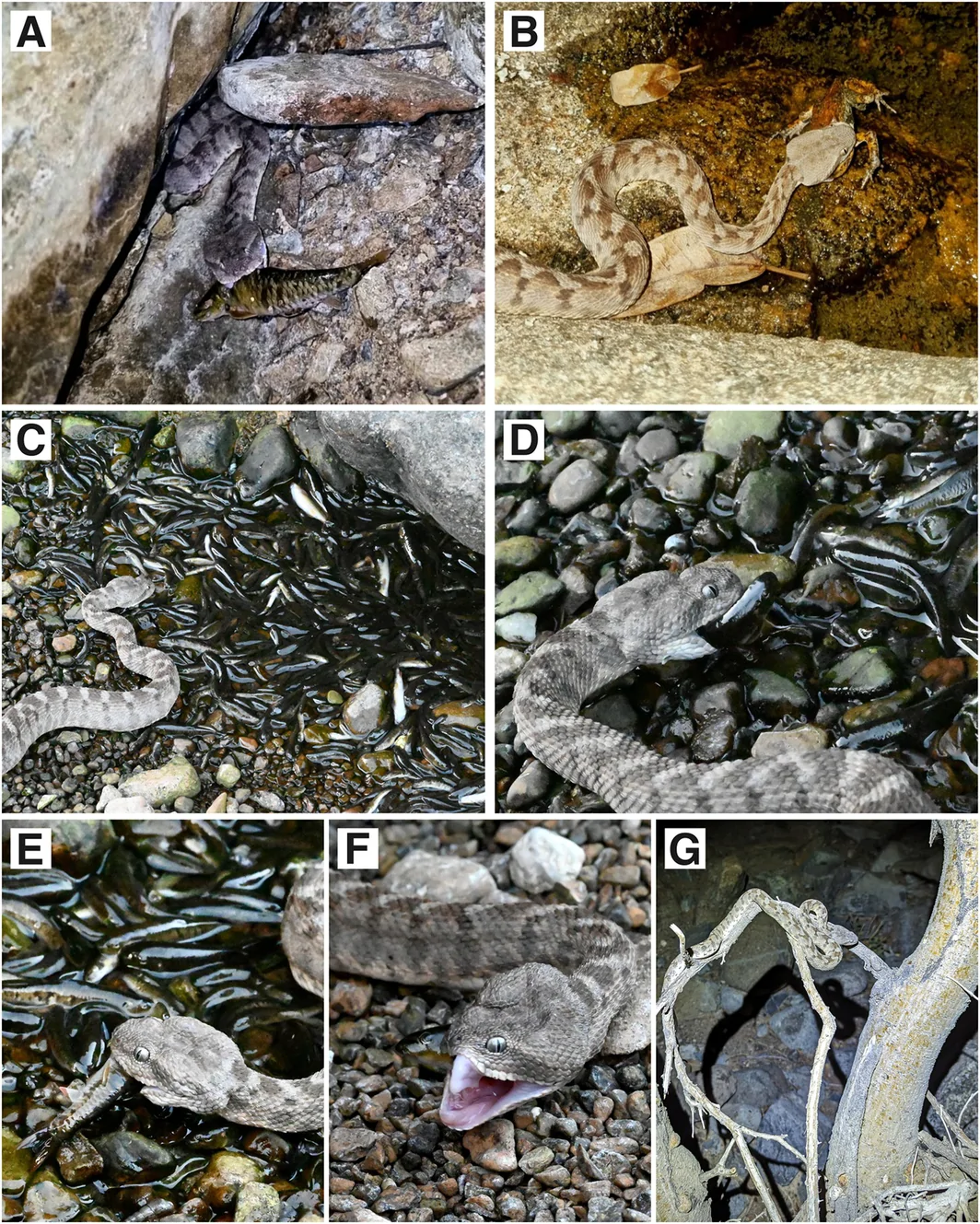
*Echis omanensis* feeding observations. (A, B) *E. omanensis* feeding on *Garra barreimiae* (A) and *Sclerophrys arabica* (B) in Wadi Shawka, United Arab Emirates. (C–F) *E. omanensis* feeding on *G. barreimiae* from a drying pool in Wadi Wurrayah, United Arab Emirates. (G) *E. omanensis* presumably in ambush on a tree trunk where it remained for Â ± 5 h after being observed for the first time.

*Observation 2*. On June 1, 2025, in Wadi Wurrayah, Fujairah, United Arab Emirates, at about 5:25 am we observed an adult *E. omanensis* approaching a drying pool filled mostly with *G. barreimiae* and some tadpoles (most likely all *S. arabica* ). We followed the viper to the pool and started taking photos and videos at 5:28 am (time stamp on the first video) when the viper first reached the fish buffet (Figure 1C). For the next 5 min the viper proceeded with frequent tongue‐flicking and occasional head jerks in response to the movement of the struggling fish. At 5:33 am the snake picked off the first small fish and held on to it for 5 s (Figure 1D), but the fish managed to escape when the viper opened its mouth to swallow it. The viper continued to search for the next fish as before. A slightly larger fish was caught at 5:36 am (Figure 1E), at which point we started recording a new video. After holding the fish for ±30 s the viper started consuming the live fish tail first. The snake was startled by our movements 2m18sec into the video recording when only the head of the fish was still outside the snake's mouth (Figure 1F). At this point we decided to leave the snake be to consume the rest of its meal without our presence.

*Garra barreimiae* are common in the wadis of the northern Hajar mountains of the United Arab Emirates and Oman where they are mostly found in small gravel pools (Freyhof et al. 2020). Their biology is not well known, but they are thought to spawn in anticipation of flooding and are known to travel upstream against the flowing current, and even short distances out of water. It has been speculated that *Garra* can survive drying wadis by retreating to lower pools under boulders or deep in the wet gravel as juveniles, yet many also get trapped when water levels drop below the gravel surface (Freyhof et al. 2020).

*Garra* may be included as an opportunistic seasonal component in the diet of *E. omanensis* , particularly for those populations inhabiting larger wadis such as Wadi Wurrayah and Wadi Shawka. These wadis may experience multiple flooding events during the winter months from October to April, which can sustain streams for several months of the year (Tourenq et al. 2011; Alsharhan and Rizk 2020). During this time, the combination of flowing water and deeper water bodies may make fish inaccessible to terrestrial predators like *E. omanensis* that are unlikely to hunt in the water. Although there may be occasional bouts of rain during the summer months from May to September, the low humidity (< 10%) and high daytime temperatures (up to 50°C) gradually dry the wadis, reducing the surface water to small streams in the deepest gorges or shaded pools. The fish that become trapped on the surface or confined to small pools then become easy prey to opportunistic terrestrial predators.

Vipers are broadly classified as ambush predators and therefore believed to have evolved to be more opportunistic, as they are likely to encounter prey less often than active foragers (Glaudas et al. 2019). Although fish and frogs are the only verifiable prey recorded for *E. omanensis* , populations at higher elevations or dry wadis are expected to feed on non‐aquatic vertebrates. We have previously observed an individual in a dry mountainous region waiting in ambush on a branch facing the tree trunk from ±9 pm when we found it to ±2 am when we passed the tree again (Figure 1G). At this location, *Ptyodactylus* geckos were often observed on the trunks of these trees and would make a suitable meal choice for *E. omanensis* . Our observations, showing *E. omanensis* actively seeking out sedentary prey in drying pools and presumably waiting in ambush in trees, form part of the growing body of literature suggesting that vipers are not strictly ambush foragers and may use a variety of hunting strategies depending on time, season, or prey types (Sorrell 2009; Kodama 2020; Subach et al. 2022).

Only one viper, *Agkistrodon piscivorus* , is considered to be truly piscivorous, where fish make up the largest component of its published diet (table 1; Holding et al. 2022). From previously assembled diet datasets, exceptionally few other vipers have been reported to feed on fish. For example, Busschau and Boissinot (2022) only identified 6 out of 224 vipers that were previously reported to feed on fish. It is important to note that these feeding records came from various sources, including field guides and books alongside primary observations. Among the published datasets based exclusively on primary sources, only *Agkistrodon piscivorus* and two other vipers, *Bothrops asper* and *Gloydius blomhoffii* , were reported to feed on fish (Grundler 2020; Holding et al. 2022). To gain better insight into fish‐eating vipers we did a targeted literature search using Google Scholar and ChatGPT to search for online sources as well as using field guides or books available to us to compile a comprehensive list of vipers reported to feed on fish. We subsequently searched for additional primary sources to verify the reported diets of each species. We considered primary sources as literature reporting direct field observations or gut contents, as well as citizen science observations of predation events on iNaturalist (www.inaturalist.org). All primary and non‐primary sources were verified to explicitly state fish‐feeding for all species summarized in Table 1 and Appendix A.

**TABLE 1:** Summarized diet data for fish‐eating viper species.

Species	Mammals	Birds	Reptiles	Amphibians	Invertebrates	Fish
*Agkistrodon bilineatus*	P	•	P	•	•	•
*Agkistrodon howardgloydi*	P	•	P	P		•
*Agkistrodon piscivorus*	P	P	P	P	P	P
	4^a^	3^a^	18^a^	17^a^	9^a^	49^a^
*Bitis nasicornis*	P	P	P	P		•
	70^a^; 81^b^	6^a^; 0^b^	1^a^; 0^b^	24^a^; 19^b^	0^a,b^	0^a,b^
*Bothrops asper*	P	P	P	P	P	P
	59^a^	3^a^	19^a^	19^a^	0^a^	0^a^
*Bothrops atrox*	P	P	P	P	P	P
	46^a,b^	3^a,b^	16^a,b^	34^a,b^	2^a,b^	0^a,b^
*Bothrops leucurus*	P	P	P	P		P
	56^a,b^	3^a,b^	28^a,b^	13^a,b^	0^a,b^	0^a,b^
*Calloselasma rhodostoma*	P	P	P	P	P	P
	54^a^	0^a^	38^a^	8^a^	0^a^	0^a^
*Craspedocephalus malabaricus*	P	•	P	P	P	P
	8^c^	0^c^	14^c^	75^c^	2^c^	2^c^
*Echis omanensis*	•	•	•	P	•	P
*Gloydius blomhoffii*	P	•	P	P	P	P
	9^a^	0^a^	6^a^	73^a^	7^a^	5^a^
*Gloydius halys*	•	•	•	•	•	•
*Gloydius tsushimaensis*	•			P		P
*Gloydius ussuriensis*	P	P	P	P	P	•
	9^d^	2^d^	35^d^	24^d^	30^d^	0^d^

*Note:* Prey classes supported by quantitative data and additional primary sources are indicated using a shaded “P” and a dot (•) indicates support from non‐primary sources (see Appendix A for sources). Values indicate percentage (%) of each prey class from the diets summarized by Holding et al. (2022)^a^ and Glaudas et al. (2019)^b^, and the calculated percentages from the data presented by Bhatnagar and Kalki (2021)^c^ and Kim and Oh (2014)^d^, indicated with the corresponding superscripts. Values rounded to the nearest percentage.

In our review we identified 14 viper species that were reported to feed on fish, and we were able to verify 9 fish‐eating species from primary sources (Table 1). Fish‐eating vipers generally include a variety of prey in their diet, a reflection of their generalist diets. Most of these species are known to hunt along streams or water bodies, which is apparent from the large proportion of amphibians in the diets of those species with quantitative diet information (Table 1). As expected, using the diet and habitat dataset from Busschau and Boissinot (2022) for 224 vipers, we detected a significant association between fish‐feeding and vipers associated with water (Fisher's Exact Test, *p* = 6.96 × 10^−5^, OR = 13.23, 95% CI [2.83, 125.21]). Two of the fish‐eating vipers, *Calloselasma rhodostoma* and *Craspedocephalus malabaricus*, are not recorded as water‐associated in this dataset (Figure 2), yet both species are associated with tropical forests where they could encounter fish in flooded forests or drying streams. In fact, the diet of *C. malabaricus* comprises mostly amphibians (Table 1), and this may lead them to water. In this regard, we detected a significant association between fish‐feeding and amphibian‐eating vipers among all species (Fisher's Exact Test, *p* = 3.99 × 10^−4^, OR = ∞, 95% CI [2.64, ∞]) and among the 77 water‐associated vipers (Figure 2, Fisher's Exact Test, *p* = 2.96 × 10^−2^, OR = ∞, 95% CI [1.09, ∞]). The association between fish‐feeding and amphibian‐eating, water‐associated vipers provides additional insight into the ecology of fish‐eating vipers and the moist/wet environments where they are likely to encounter fish.

**FIGURE 2:**
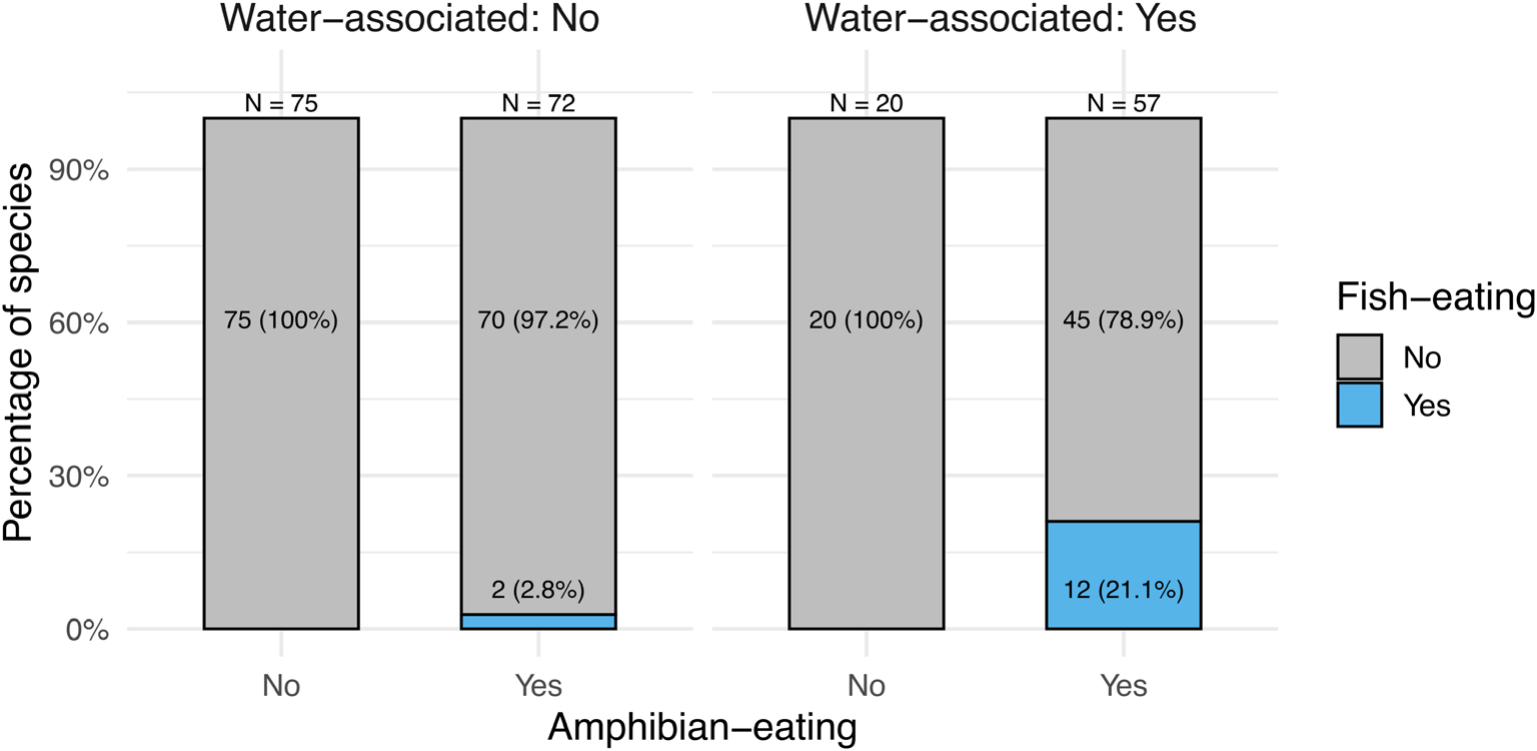
Niche association among fish‐eating viper species. Showing the percentage of species associated with water and the percentage of species reported to feed on amphibians and fish. Fisher's exact tests retrieved significant associations (*p* < 0.05) between fish predation and water‐associated species and between fish predation and amphibian‐eating species across all 224 viper species in the habitat and diet dataset from Busschau and Boissinot (2022).

We initially speculated that the predation on fish in our *E. omanensis* observations may have been accidental, given the scent of frogs in the pools where the snakes were observed hunting, or that the similar scent of fish and frogs in the same pool may entice the snakes to eat fish. It has, however, been shown that *Echis coloratus* , the sister species to *E. omanensis* , does not select ambush sites based on scent (Tsairi and Bouskila 2004), and the piscivorous *A. piscivorus* does not exhibit preference for fish or mice prey based on scent cues (Lutterschmidt et al. 2024). This makes the scent hypothesis quite unlikely and instead supports that vipers are generalists and opportunistic predators, again alluding to the hypothesis that dietary composition is determined by the availability/accessibility of prey in the environments where these vipers are hunting and not necessarily prey preference.

Vipers and other opportunistic ambush foraging snakes consume a wide variety of prey as well as a wide range of prey sizes, occasionally eating much larger prey (Glaudas et al. 2019). As seen in our fish‐eating observations, vipers may also occasionally eat very small prey items. Herein lies the caveat that small prey items like fish may be digested much faster than larger bonier prey, like mammals or even frogs. Since much of what we know about viper diets comes from studying gut contents, it is possible that small rapidly digested prey items are underrepresented in gut‐content studies relative to their actual consumption frequency, making field observations particularly important for detecting such prey. Although gut contents are often the preferred source of dietary information for snakes because they can generate more data points relatively quickly compared to observational data, gut‐content studies can suggest a significantly more specialized diet (Barends et al. 2024). This is apparent in Table 1 where fish were not detected in gut‐content studies although there are primary sources verifying fish in the diets of these vipers. It is possible that vipers feed on fish more often than realized (although still rarely).

The detectability of fish in the diets of vipers can be influenced by regional, ontogenic or seasonal differences in diet composition (Daltry et al. 1998; Kim and Oh 2014; Kodoma 2019; Akdağ and Çiçek 2024). Perhaps the most compelling evidence of the latter is in the unpublished study on *Gloydius tsushimaensis* suggesting a shift to fish prey in the summer compared to the amphibian dominated diet in spring and autumn (Kodoma 2019). Similarly, fish were not detected in the diet studies on the *Bothrops* species (Table 1), yet all three species were observed feeding on eels suspected to be migrating across the seasonally flooded forests where these vipers occur (Oliveira and Martins 2003; De Freitas and Loebmann 2010; Díaz‐Ricaurte 2018). Although we could not verify fish in the diets of *Gloydius halys* or *Bitis nasicornis*, it is likely that fish‐eating may be region‐specific. *Gloydius halys*, like *E. omanensis* , is largely adapted to arid environments where it is associated with mountainous water bodies, but populations from eastern Mongolia were believed to be especially mesophilic and feed on frogs and fish (Orlov et al. 2002). Spawls et al. (2002) described the diet of *Bitis nasicornis* as including mostly mammals but also referred to reports of amphibians and fish being taken. Spawls et al. (2022), however, omitted fish given that the original inclusion of fish came from Pitman (1974) who described frogs, toads, and even fishes to be included in the diet of *Bitis nasicornis* according to locality, especially west Africa, yet the source of this information was uncertain as fish were not mentioned in the associated references (Spawls, personal communication). Furthermore, we are aware that our list of fish‐eating vipers is likely not exhaustive despite our best efforts. We noted that several of our sources, both primary and non‐primary, were published more than two decades ago (Appendix A). It is possible that more fish‐eating vipers were historically recorded, but that information became lost in time, forgotten in older literature that is inaccessible online today.

Our investigation into the diet of *E. omanensis* and other fish‐eating vipers highlights some of the challenges in understanding the complexity of snake diets (see Maritz et al. 2021). Observing snakes feeding in the wild is rare. It is even more rare to observe snakes feeding on uncommon, seasonal or locality‐specific prey items like fish. Knowledge of these prey items may be region‐specific, for example, confined to regional field guides, unpublished local knowledge, or be hidden in older literature that may be especially difficult to access. We therefore emphasize the importance in sharing natural history observations in the form of natural history notes, on citizen science platforms such as iNaturalist (www.inaturalist.org) or social media (Maritz and Maritz 2020; Bhatnagar and Kalki 2021) even when the diet of a species is assumed to be known.

## Author Contributions

**Theo Busschau:** conceptualization (lead), data curation (lead), formal analysis (lead), funding acquisition (supporting), investigation (equal), visualization (lead), writing – original draft (lead), writing – review and editing (equal). **Arabella Willing:** conceptualization (lead), funding acquisition (equal), investigation (equal), writing – review and editing (equal). **Borja de las Heras:** conceptualization (supporting), investigation (equal), writing – original draft (supporting), writing – review and editing (equal). **Rami Khashab:** conceptualization (supporting), investigation (equal), writing – original draft (supporting), writing – review and editing (equal). **Abdul Nasser Obeidat:** funding acquisition (equal), project administration (equal), validation (lead), writing – review and editing (equal). **Altaf Habib:** funding acquisition (equal), project administration (equal), validation (supporting), writing – review and editing (equal). **Andrew Gardner:** conceptualization (lead), funding acquisition (equal), investigation (equal), project administration (equal), writing – original draft (supporting), writing – review and editing (equal).

## Conflicts of Interest

The authors declare no conflicts of interest.

## Data Availability

All associated data are presented within the manuscript in Figure 1, Table 1, and Appendix A.

## Appendix A Sources of Additional Diet Information by Species

***Agkistrodon bilineatus***

Non‐Primary sources:

Campbell JA, Lamar WW. 2004. The venomous reptiles of the western hemisphere, Volume 1. Comstock: Cornell University Press.

Primary sources:

Nieto‐toscano LF, martínez‐coronel M. 2021. *Agkistrodon bilineatus* (Common Cantil). Diet. Herpetol Rev. 52(2):414.

David Jacobo, some rights reserved (CC BY‐NC; https://creativecommons.org/licenses/by‐nc/4.0).

Unmodified from iNaturalist (https://www.inaturalist.org/observations/136984291).

***Agkistrodon howardgloydi***

Non‐primary sources:

*Agkistrodon howardgloydi* ‐ Wikipedia. [accessed 2026 Mar 3]. https://en.wikipedia.org/wiki/Agkistrodon_howardgloydi.

Primary sources:

Solorzano A, Romero M, Maria Gutierrez J. 1999. Venom composition and diet of the Cantil *Agkistrodon bilineatus howardgloydi* (Serpentes: Viperidae). 44(4):478–483.

***Bitis nasicornis***

Non‐Primary sources:

Spawls S, Howell K, Drewes RC, Ashe J. 2002. Field guide to the reptiles of East Africa: Kenya, Tanzania, Uganda, Rwanda and Burundi. London: Academic Press.

***Bothrops asper***

Primary sources:

Díaz‐Ricaurte JC. 2018. First record of attempted piscivory by *Bothrops asper* (Garman, 1883) (Squamata, Viperidae) on a swamp eel, genus *Synbranchus*. Herpetol Notes. 11:835–837.

Loaiza‐Lange A, Székely D, Torres‐Carvajal O, Tinoco N, Salazar‐Valenzuela D, Székely P. 2023. Feeding ecology of the Terciopelo pit viper snake (*Bothrops asper*) in Ecuador. PeerJ. 11:e14817.

***Bothrops atrox***

Primary sources:

Oliveira ME, Martins M. 2003. *Bothrops Atrox* (Common Lancehead). Prey. Herpetol Rev. 34(1).

***Bothrops leucurus***

Primary sources:

de Freitas MA, Loebmann D. 2010. Bothrops leucurus (Jararaca/Bahia Lancehead). Diet. Herpetol Rev. 41(2):234.

***Calloselasma rhodostoma***

Primary sources:

Daltry JC, Wüster W, Thorpe RS. 1998. Intraspecific variation in the feeding ecology of the crotaline snake *Calloselasma rhodostoma* in Southeast Asia. Journal of Herpetology. 32(2):198–205.

Vncreatures, some rights reserved (CC BY‐NC; https://creativecommons.org/licenses/by‐nc/4.0).

Unmodified from iNaturalist (https://www.inaturalist.org/observations/290323119).

***Echis omanensis***

Non‐primary sources:

Egan D. 2008. Snakes of Arabia: a field guide to the snakes of the Arabian Peninsula and its shores. Motivate Publishing.

Burriel‐Carranza B, Els J, Carranza S. 2022. Reptiles & Amphibians of the Hajar Mountains. Madrid: Consejo Superior de Investigaciones Científicas.

***Gloydius blomhoffii***

Non‐primary sources:

Mamushi ‐ Wikipedia. [accessed 2026 Mar 4]. https://en.wikipedia.org/wiki/Mamushi.

***Gloydius halys***

Non‐primary sources:

Orlov N, Natalia A, Barabanov A, Ryabov S, Khalikov R. 2002. Diversity of vipers (Azemiopinae, Crotalinae) in East, Southeast, and South Asia : Annotated checklist and natural history data (Reptilia: Squamata: Serpentes:Viperidae). Faunistische Abhandlungen des Staatlichen Museums für Tierkunde Dresden. 23(812):177–218.

Jablonski D, Koleska D. 2017. *Eremias stummeri* (Squamata: Lacertidae) as a prey for *Gloydius halys* complex (Serpentes: Viperidae) from Kyrgyzstan. Phyllomedusa. 16(1):121–124.

***Gloydius tsushimaensis***

Non‐primary sources:

*Gloydius tsushimaensis* ‐ Wikipedia. [accessed 2026 Mar 5]. https://en.wikipedia.org/wiki/Gloydius_tsushimaensis.

Primary sources:

Kodama T. 2020. *Gloydius tsushimaensis* (Tsushima Island Pitviper). Diet and scavenging. Herpetological Review. 51(2):351.

Kodoma T. 2019. Feeding ecology of a fish‐eating pitviper, *Gloydius tsushimaensis*: seasonal change of foraging sites [Conference abstract]. Biology of pitvipers 3. Rodeo, New Mexico.

***Gloydius ussuriensis***

Non‐primary sources:

Orlov N, Natalia A, Barabanov A, Ryabov S, Khalikov R. 2002. Diversity of vipers (Azemiopinae, Crotalinae) in East, Southeast, and South Asia : Annotated checklist and natural history data (Reptilia: Squamata: Serpentes:Viperidae). Faunistische Abhandlungen des Staatlichen Museums für Tierkunde Dresden. 23(812):177–218.

***Craspedocephalus malabaricus***

Non‐primary sources:

Gawas M, Rane S, Sawant N. 2023. A review of Malabar pit viper, *Craspedocephalus malabaricus* (Jerdon 1854), ecology from the Western Ghats of India and notes on feeding behavior. Reptiles & Amphibians. 30(1):e18085.
